# Remarkable recovery from a *Cladophialophora bantiana* fungal brain abscess in an immunocompromised patient: a case report

**DOI:** 10.1128/asmcr.00150-25

**Published:** 2026-01-15

**Authors:** Molly S. Walkenhorst, Elizabeth Savage, Kellie Wark, Joanna Kimball, Rachel Weihe, Kathryn Lamberton, Robert Hamilton-Seth, Julia D. Hankins, Nelli S. Lakis

**Affiliations:** 1 Department of Pathology and Laboratory Medicine, The University of Kansas Medical Center, Kansas City, Kansas, USA; 2 Department of Infectious Disease, The University of Kansas Medical Center, Kansas City, Kansas, USA; Vanderbilt University Medical Center, Nashville, Tennessee, USA

**Keywords:** case report, phaeohyphomycosis, fungal brain abscess, *Cladophialophora bantiana*

## Abstract

**Background:**

*Cladophialophora bantiana* is a globally distributed dematiaceous mold which acts as an uncommon source of human infection. With a predilection for the central nervous system (CNS), this organism accounts for nearly half of all cerebral phaeohyphomycosis, with a mortality rate over 60%. Due to the rarity of this disease, treatment regimens are primarily based on retrospective reviews of case reports. The best clinical response occurred with a dual approach of surgical resection and antifungal therapy, though mortality rates remain high.

**Case Summary:**

A 68-year-old immunocompromised woman presented with imaging findings concerning for a neoplastic process, such as a glial neoplasm or CNS lymphoma. With microbiology, flow cytometry, and cytology of the cerebrospinal fluid negative and frozen section of the lesion favoring glial neoplasm, the scarce pigmented fungal hyphae on permanent sections were unexpected. Broad-range fungal DNA testing identified the organism as *C. bantiana*. Without further surgical resection, dual antifungal therapy was utilized to achieve clinical stability and return to pre-infection baselines.

**Conclusion:**

This is a rare case of a *C. bantiana* fungal brain abscess in an immunocompromised patient. Complete surgical resection was not possible; however, this case provides additional data to show the benefits of a dual antifungal treatment approach without additional surgical intervention, and the dosages used to achieve stability at 18 months post-presentation. This case highlights the importance of prompt efforts in determining causative organisms in unexpected fungal brain abscesses, which allows for targeted and improved success in the treatment of this highly fatal disease.

## INTRODUCTION

Dematiaceous molds are ubiquitous environmental pigmented fungi capable of causing a spectrum of clinical diseases, from soft tissue nodules to disseminated infections. *Cladophialophora bantiana* is a globally distributed dematiaceous mold characterized by the production of dihydronaphthalene melanin within its cell wall (1, 2).

Although an uncommon cause of human infections, *C. bantiana* exhibits neurotropism, accounting for 48% of cerebral phaeohyphomycosis, with a typical presentation of solitary or multiple brain abscesses and a mortality rate exceeding 60% regardless of immune status (1–4). It has been proposed that *C. bantiana* arrives at the central nervous system (CNS) through hematogenous dissemination from primary pulmonary or skin lesions (1, 3). Unlike other neurotropic fungi, which predominantly affect immunocompromised individuals, *C. bantiana* is notable for its occurrence in immunocompetent hosts (2–5). Symptoms at presentation typically reflect increased intracranial pressure (1–3). Radiographically, *C. bantiana* cerebral abscesses lack pathognomonic features and are not often considered in initial differential diagnoses.

Published treatment methods have varied over the years, with current methods focusing on complete surgical resection of the fungal abscess and systemic single or multiple antifungal regimens. Without treatment, *C. bantiana* cerebral phaeohyphomycosis is almost universally fatal (2, 3). Unimodal approaches with either surgery or antifungal therapy have historically had high fatality rates. The most favorable outcomes are reported in cases utilizing combined surgical and antifungal therapy; however, even with this dual-modality approach, mortality remains high, with approximately 56.7% of cases resulting in death (2, 4). Given this infection’s aggressive nature, early recognition is critical for prompt initiation of targeted antifungal therapy. The primary treatment goals are to achieve clinical stabilization and radiographic resolution or reduction of intracerebral lesions.

We report the case of a 68-year-old immunocompromised female whose initial presentation and findings were highly suggestive of a primary glial neoplasm. However, broad-range fungal DNA detection using polymerase chain reaction (PCR) performed on paraffin-embedded biopsied brain tissue identified the lesion as *C. bantiana*. Although further surgical intervention was not feasible, the patient was successfully treated with long-term systemic antifungal therapy.

## CASE PRESENTATION

**Fig 1:**
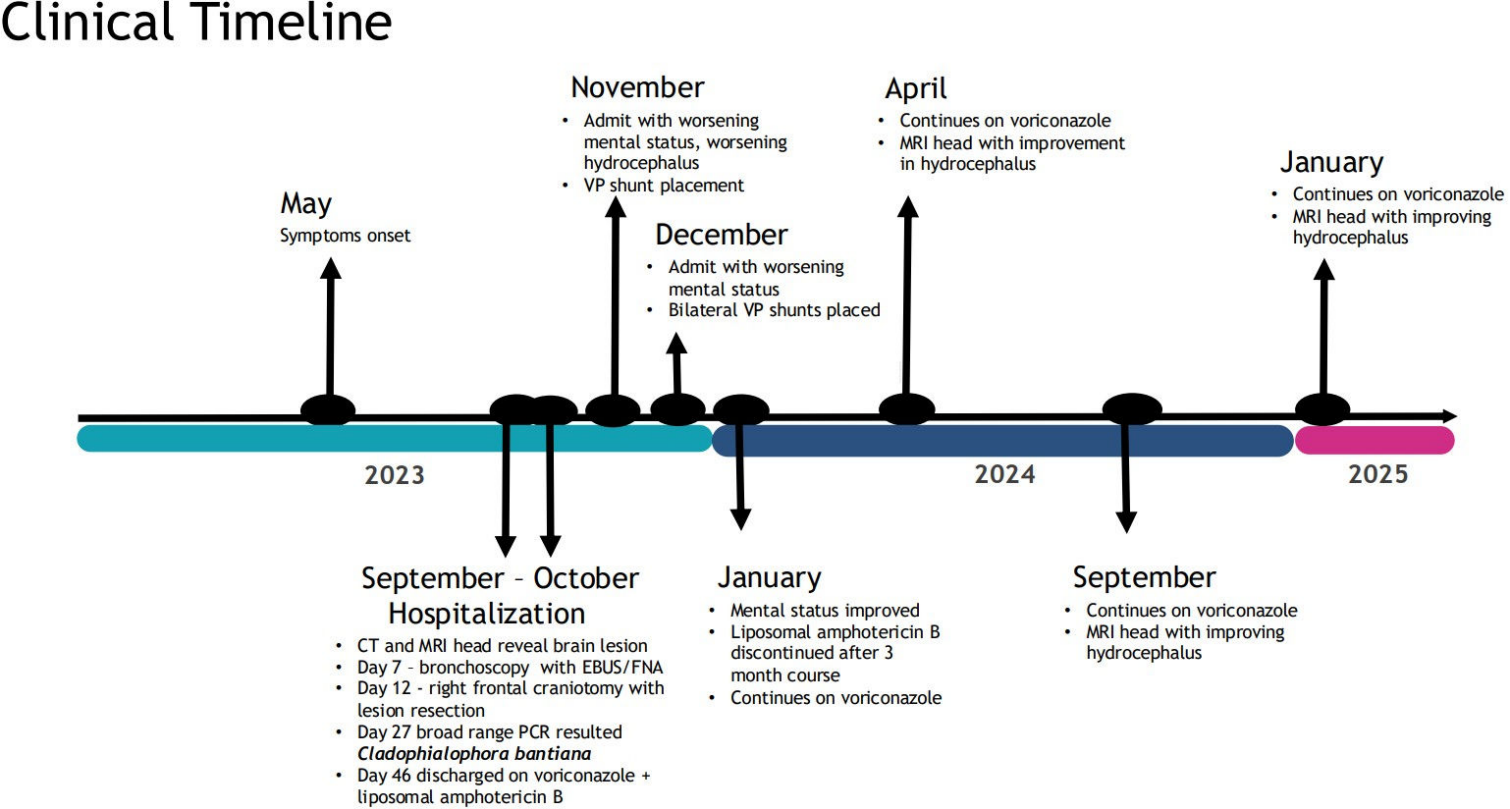
Timeline of the patient’s clinical course from onset of symptoms to current disposition. EBUS, endobronchial ultrasound. FNA, fine needle aspiration.

**Fig 2:**
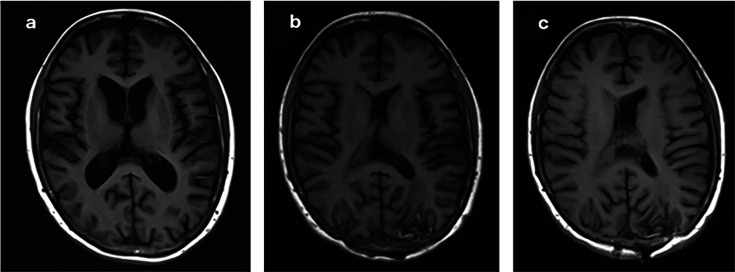
Serial MRIs of the patient’s midline mass centered between the bodies of the lateral ventricles with findings most concerning for a neoplastic process: (**a**) MRI from September 2023; (**b**) MRI from September 2024; (**c**) MRI from January 2025.

A 68-year-old woman with a history of rheumatoid arthritis on hydroxychloroquine, fibromyalgia on pregabalin, asthma, and a remote history of endometrial cancer status post-hysterectomy presented in September 2023 with 4 months of generalized malaise, nausea, unintentional 20-pound weight loss, and neurologic decline. Figure 1 shows the timeline of the patient’s clinical course. Computed tomography (CT) of the head showed a 2.7 × 1.8 × 1.7 cm ill-defined, low-density mass-like area located between and abutting the lateral ventricles, prompting admission for further work-up. Magnetic resonance imaging (MRI) of the head (Fig. 2a) showed a heterogeneously enhancing midline mass centered between the bodies of the lateral ventricles with findings most concerning for a neoplastic process, such as infiltrating glioma or CNS lymphoma. Cerebral spinal fluid (CSF) studies from a lumbar puncture showed glucose 33 mg/dL (reference: 40–75 mg/dL), protein 298 mg/dL (reference: 15–45 mg/dL), white blood cells 239/uL (30% neutrophils, 56% lymphocytes, 14% monocyte/histiocyte), red blood cells 0/uL, routine and fungal cultures negative, lymphocytosis on cytology, and a negative immunophenotypic study by flow cytometry. Two hypermetabolic right lung nodules, bilateral hilar lymph nodes, and a brain lesion were noted on positron emission tomography/CT. Fine needle aspiration of a lymph node from a bronchoscopy with endobronchial ultrasound guidance was negative for malignancy, with scant polymorphous lymphocytes and abundant anthracotic macrophages present. No evidence of infection was noted on smears or cell block. Bronchoalveolar lavage was not performed, and no biopsies were sent for microbiologic assessment.

**Fig 3:**
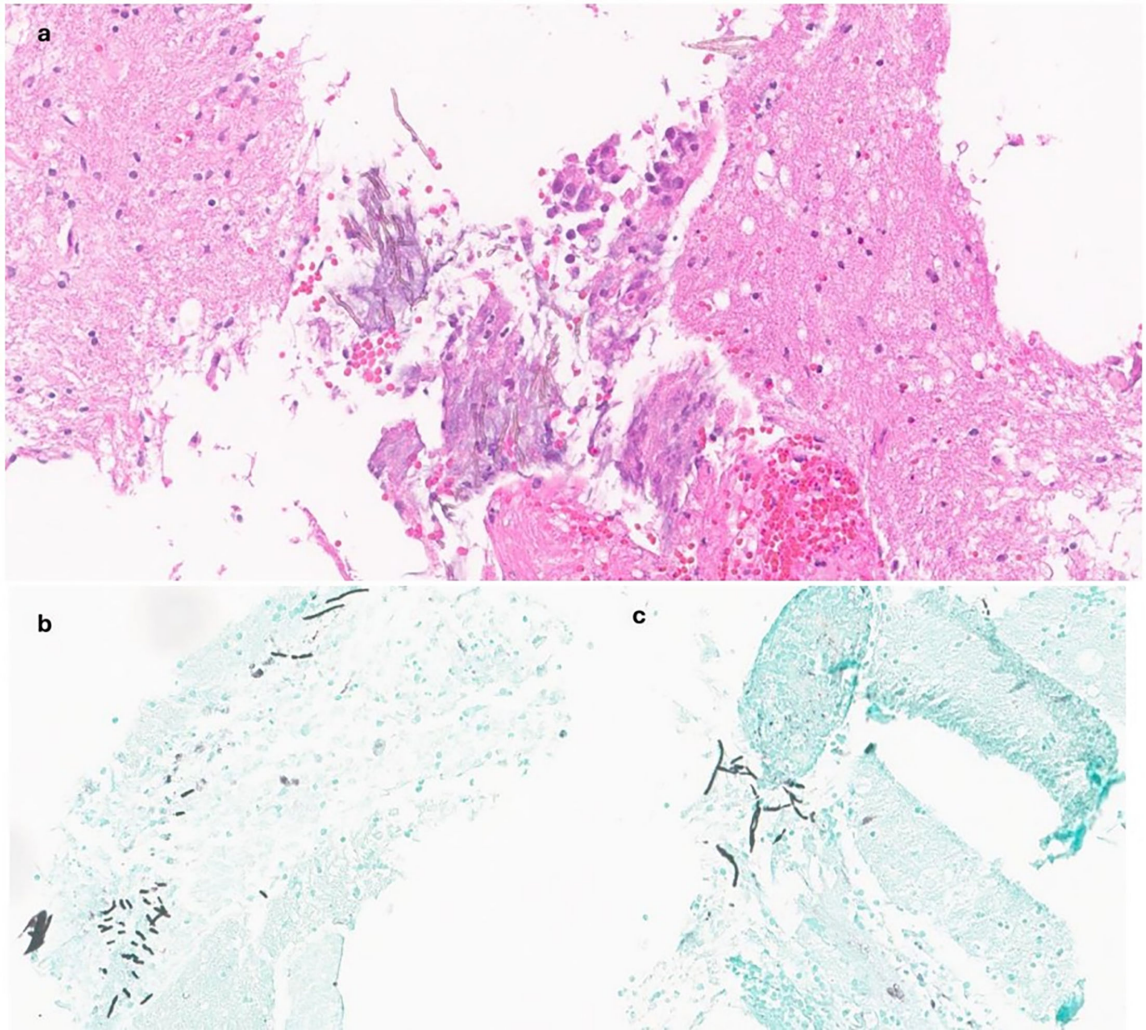
Corpus callosum lesional brain biopsy permanent sections findings with (**a**) H&E at 20× showing fungal elements and (**b** and **c**) GMS at 20× highlighting hyphal elements.

On hospital day 12, she underwent a right frontal craniotomy with partial resection of the corpus callosum lesion. Intraoperative frozen section assessment from the lesion was reported as glial lesion, favoring glial neoplasm. No microbiological cultures were obtained due to low suspicion from frozen and clinical findings. Permanent histologic examination showed fibrinopurulent exudate with scant fungal hyphae seen on both H&E and GMS stains (Fig. 3a through c). The critical finding of brown fungal hyphae concerning for dematiaceous mold was communicated to the clinical team on hospital day 14. Additional workup confirmed a lack of glial or lymphoid neoplasm. Though fungal elements were rare and paraffin-embedded tissue was depleted, broad-range fungal PCR with reflex to Sanger sequencing was attempted at the University of Washington. Results were reported as positive for fungal 28S rDNA and subclassification identifying *C. bantiana* on hospital day 26.

The Infectious Diseases team was consulted on hospital day 15 and started empiric intravenous liposomal amphotericin B 5 mg/kg daily with pre-post intravenous normal saline, while hydroxychloroquine was held, based on suspicion for dematiaceous mold infection. Further resection of the brain lesion was not feasible due to the anticipated risk of significant neurologic morbidity and mortality. After approximately 2 weeks of daily liposomal amphotericin B at 5 mg/kg, *C. bantiana* was identified, leading to the addition of voriconazole 400 mg through nasogastric tube twice daily (10 mg/kg/dose) and an increase of liposomal amphotericin B to 6 mg/kg daily. Given the severity of the infection and due to the patient’s remarkable renal tolerance, the liposomal amphotericin B dose was increased to 7 mg/kg daily after 2 days of 6 mg/kg. Voriconazole dosing was adjusted based on therapeutic drug monitoring, with the patient’s initial voriconazole trough level measuring 5 mcg/mL, which led to a continuation of 400 mg per oral (PO) twice daily. The patient was discharged to a long-term acute care facility on liposomal amphotericin B 7 mg/kg daily and voriconazole 400 mg PO twice daily.

Over the next 2 months, the patient was admitted twice with encephalopathy related to obstructive hydrocephalus, culminating in three extraventricular drain (EVD) placements. After a 2-month dual-antifungal course, with repeat CSF cultures negative and a nearly resolved CSF pleocytosis, the temporary EVDs were internalized, and a ventriculoperitoneal shunt (VPS) was placed. On the final re-admission, a voriconazole trough level of 1.6 mcg/mL was obtained while the patient was receiving 400 mg PO twice daily (9 mg/kg/dose). A repeat trough level 1 week later was 1.4 mcg/mL, which led to a dose escalation to 450 mg PO twice daily (10 mg/kg/dose). This dose was maintained for the next several months until the next voriconazole trough level decreased to 1.3 mcg/mL, prompting another dose escalation to 500 mg PO twice daily (15 mg/kg/dose). After 1 week at the increased dose, a supratherapeutic voriconazole trough level of 6 mcg/mL was reported, resulting in a dose reduction to 450 mg PO twice daily (13 mg/kg/dose). This new dose resulted in a therapeutic voriconazole trough level of 2.5 mcg/mL.

Liposomal amphotericin B was continued for the first 3 months, maintained at 7 mg/kg thrice weekly; thereafter, voriconazole monotherapy was continued. An MRI obtained 1 year from her initial presentation (Fig. 2b) showed the persistent lobulated collection in the posterior bodies and atria of the lateral ventricles, consistent with necrotic material and fungal abscess, and improved hydrocephalus with ventricular catheters in place. Clinically, she had remarkable improvement with return to a pre-infection level of independence in activities of daily living and cognitive functioning. The patient remains stable with MRI monitoring 18 months after presentation (Fig. 2c), with VPS retained due to persistent hydrocephalus. The voriconazole dose was further adjusted based on trough levels to 300 mg twice daily (7 mg/kg/dose) without any noted toxicities during a follow-up outpatient visit the following February, with plans to continue voriconazole indefinitely, given incomplete resolution of infection on imaging in the setting of VPS placement. Having improved to baseline, she was cautiously cleared for a lower-immune-suppressing agent for her progressive rheumatoid arthritis.

## DISCUSSION

This case highlights the successful treatment of an immunocompromised patient with an intracerebral abscess caused by *C. bantiana*. This patient’s initial presentation was initially concerning for a primary brain neoplasm, a pitfall experience in other case reports of *C. bantiana* brain abscesses (5–7). CSF fungal cultures were consistently negative, similar to previously reported cases (2, 7). Though scarce, fungal hyphae on permanent sections of the sampled intracerebral mass were sufficient for broad-range fungal testing, leading to the final diagnosis of *C. bantiana* fungal intracerebral abscess and a tailored treatment. Despite incomplete surgical debridement, dual antifungal treatment, followed by continued long-term voriconazole monotherapy, was successful in treating this patient’s infection, allowing her to return to her pre-infection baseline.

Both cutaneous and pulmonary routes of infection have been proposed in the literature. In this case, the patient may have had a pulmonary nidus based on the hypermetabolic pulmonary nodules and hilar lymphadenopathy. Due to a lack of microbiological testing of the pulmonary nodules, this cannot be confirmed. Additionally, the possible resolution of these lesions cannot be assessed, as no further chest imaging has been conducted since the patient initiated antifungal treatment.

**Fig 4:**
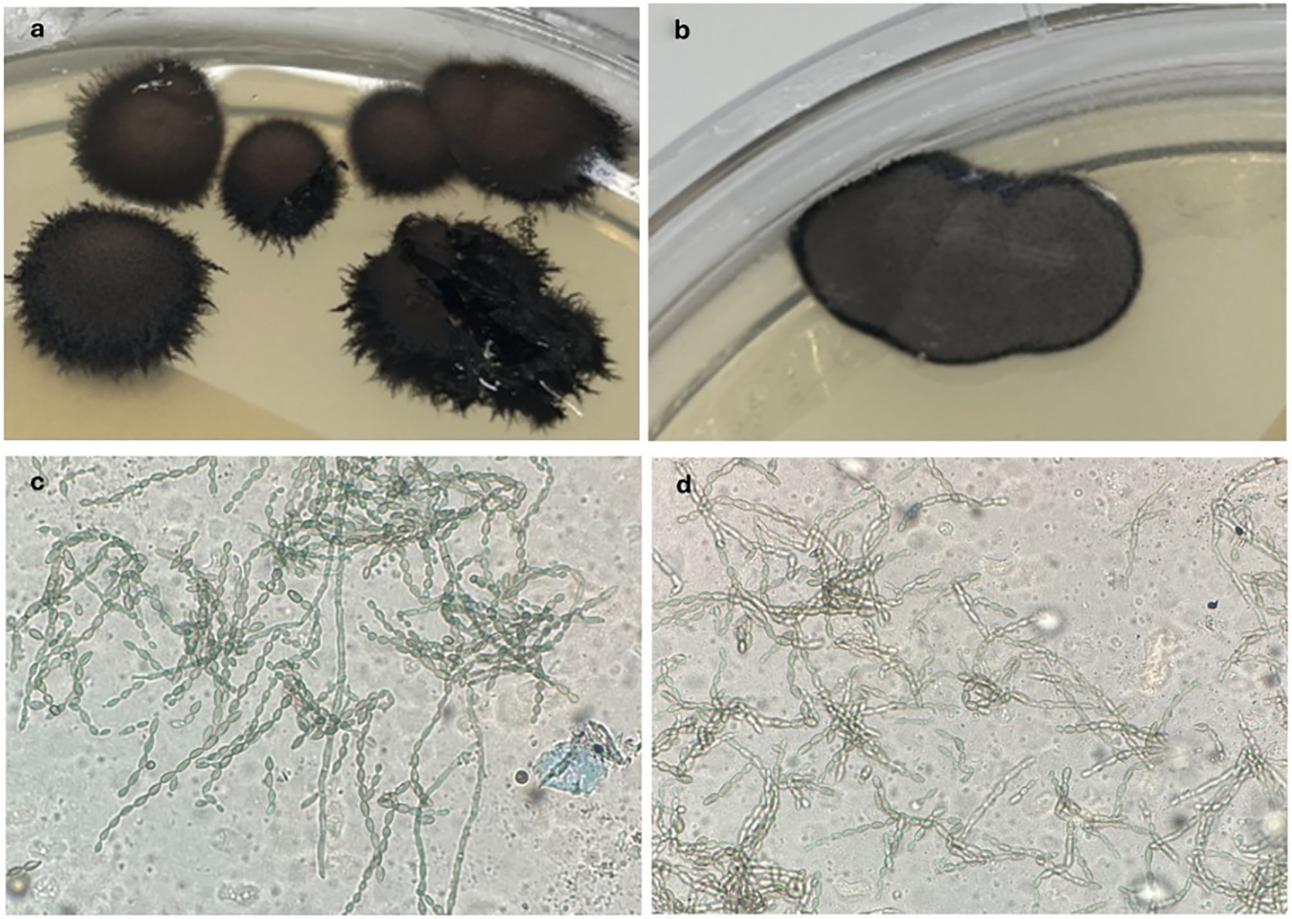
Characteristic plate (**a and b**) and lactophenol cotton blue morphology (**c and d**) of *C. bantiana*.

When the organism is grown in fungal culture, it appears as a dark-gray-to-black colony (Fig. 4a and b). It grows well on routine mycological agar. It is tolerant of cycloheximide and grows at 40°C (2, 8). Under the microscope, long, poorly branched chains of dry, ellipsoid conidia are characteristic (Fig. 4c and d) (8). Though cultures of the CSF were unsuccessful in this case and no tissue was obtained for culturing, paraffin-embedded tissue of the corpus callosum lesion was sent to the University of Washington to complete Fungal DNA detection by PCR with reflex to Sanger sequencing. This test amplifies the 28S rDNA gene of fungal organisms to first detect the presence of fungal DNA, then reflexes to Sanger sequencing of the 28S rRNA gene and the internal transcribed spacer (ITS) regions to provide identification at the species level. The ITS are highly variable regions within the rDNA array, which are the most used genetic barcode for identifying fungi to the species level (9, 10). The *Ascomycota* phylum, in which *C. bantiana* is a member, has slightly lower success rates for identification by ITS (overall probability of 0.75) than other groups, such as *Basidiomycota* (0.79) (10).

Due to its rarity, most information on treatment options originates from single case reports or short collections of cases, making systematic interpretations of these data for clinical application challenging. Few retrospective studies have been conducted, such as those completed by Revankar et al. in 2004 and Kantarcioglu et al. in 2017. The best treatment methods identified in these studies utilize a combination of surgical resection and antifungal therapy. However, mortality approaches 60%, even in these dual-approach cases. Though data for assessing this rare infection is purely retrospective, in 2021, the European Confederation of Medical Mycology, the International Society for Human and Animal Mycology, and the American Society for Microbiology gathered to create guidelines for rare mold infections, including for *C. bantiana* (11). Their recommendations conclude that liposomal amphotericin B alone or in combination with an azole and/or echinocandin or voriconazole monotherapy is moderately supported as first-line therapy. 5-Fluorocytosine can be used as an additional measure, with marginally supportive data. The exact extent of surgical resections and length of antifungal treatment necessary for improved outcomes remains unknown, though this case demonstrates that incomplete excision with liposomal amphotericin B combined with voriconazole, followed by long-term voriconazole treatment, can lead to a successful resolution.

## Data Availability

The identified sequences from this case have been submitted to GenBank. The 28S sequence can be found under accession number PX490376. The ITS sequence can be found under accession number PX577151.
